# Hippocampal-striatal interaction in Parkinson’s disease with mild cognitive impairment

**DOI:** 10.1016/j.prdoa.2026.100443

**Published:** 2026-04-18

**Authors:** Jingjing Xu, Sijia Tan, Xiaohui Zhang, Minming Zhang, Xiaojun Xu

**Affiliations:** a Department of Radiology, The Second Affiliated Hospital, Zhejiang University School of Medicine, Hangzhou, China; b Department of Nuclear Medicine and PET Center, The Second Affiliated Hospital, Zhejiang University School of Medicine, Hangzhou, Zhejiang, China

**Keywords:** Cognitive impairment, Neuroimaging, Hippocampus, Striatal dopamine, Asymmetry index

## Abstract

**Background:**

Striatal dopaminergic dysfunction and hippocampal degeneration contribute to cognitive impairment in Parkinson’s disease (PD). However, their interaction in PD-related cognitive decline remains unclear.

**Objective:**

We aimed to investigate whether asymmetry of striatal dopaminergic integrity and hippocampal subfield volume are associated with cognitive performance in PD, and whether their statistical interaction provides additional explanatory value.

**Methods:**

A cohort of 53 participants from the Parkinson’s Progression Markers Initiative (PPMI) was analyzed, including 33 PD with mild cognitive impairment (PD-MCI) patients and 20 PD with normal cognition (PD-NC) patients. All patients had complete 18F-AV-133 vesicular monoamine transporter 2 PET and 3D-T1w imaging. Asymmetry indices (AI) were calculated for striatal 18F-AV-133 standard uptake volume ratio (SUVR) and hippocampal subfield volumes.

**Results:**

PD-MCI showed higher AI in caudate and anterior putamen uptake versus PD-NC. Hippocampal CA2/3 and granule cell layer AI correlated with MoCA. Caudate and anterior putamen uptake AI linked to multiple cognitive domains. Regression analysis revealed interactions between anterior putamen SUVR AI and CA2/3 AI on MoCA in PD (p = 0.039), and posterior putamen SUVR AI with CA2/3 AI on MoCA in PD-MCI (p = 0.002).

**Conclusion:**

Striatal dopaminergic asymmetry and hippocampal subfield asymmetry are both associated with cognitive impairment in PD. In exploratory regression models, the association between CA2/3 asymmetry and global cognition varied according to the degree of striatal dopaminergic asymmetry. These findings clarified the relationship between hippocampus and dopaminergic pathways in PD with cognitive decline, and deepen understanding of the pathophysiological mechanisms of cognitive impairment.

## Highlights

• PD-MCI shows higher asymmetry in striatal dopaminergic uptake versus PD-NC.
• Hippocampal CA2/3 and granule cell layer AI correlated with MoCA.
• Striatal and hippocampal asymmetry interact to impact cognition in PD.

## 1 Introduction

Parkinson’s disease (PD) is a progressive neurodegenerative disorder primarily characterized by motor dysfunction, resulting from dopamine neurodegeneration in the nigrostriatal pathway [1]. In addition to motor symptoms, up to 42.5% of patients have cognitive impairment at initial diagnosis of PD [2]. PD with mild cognitive impairment (PD-MCI) present early cognitive impairment without impact on daily function, and have a higher risk of developing dementia [3]. Although the precise role of the hippocampus in cognitive function in Parkinson's disease is not yet comprehensively understood, the hippocampus has been reported as the cornerstone for cognitive deficits in PD [4]. Moreover, dysfunction in the nigrostriatal dopamine neurotransmitter system has been linked to cognitive impairment in PD [5], suggesting a potentially complex interplay between hippocampal and dopaminergic systems in developing cognitive decline in PD [6].

Emerging evidence from animal models and neuroimaging studies highlights the intricate relationship between the hippocampus and dopaminergic pathways in cognitive function [7], [8], [9]. Previous studies using neural fiber tract tracing techniques have confirmed a connection between the hippocampus and a key node of the dopaminergic pathway—the ventral tegmental area [7]. Dopamine D1 receptors are expressed in the molecular layer of the hippocampus, enabling dopamine to modulate intrinsic membrane properties and synaptic transmission of the hippocampus [8]. In healthy people, a functional magnetic resonance imaging (MRI) study showed that activation of the dopaminergic midbrain can enhance hippocampus-dependent memory function [10]. Therefore, the interaction between the hippocampus and the dopaminergic pathway plays an important role in cognitive function. Despite these findings, the underlying mechanisms through which hippocampal and dopaminergic dysfunction interact to contribute to cognitive impairment in PD remained unclear.

Pathologically, PD is characterized by the abnormal aggregation of α-synuclein, which leads to dopaminergic neuronal degeneration [11]. Notably, α-synuclein deposits are not confined to the nigrostriatal region but are also found in the hippocampus of the limbic system [12]. Electrophysiological studies indicate that α-synuclein accumulation in the hippocampus leads to structural damage, abnormal excitability, and impaired synaptic transmission, ultimately disrupting hippocampus-dependent memory functions [13], [14]. Furthermore, a neuroimaging study demonstrated that non-demented PD exhibited hippocampal activation as compensatory mechanism to striatal damage during executive tasks [15], whereas such activation was absent in PD-MCI [16]. Therefore, a cooperative mechanism involving hippocampal-striatal interactions may be involved in cognitive decline in PD. However, most of the studies focused on the interaction effect on cognition between overall hippocampal function and striatal dopaminergic pathways, but not hippocampal subregions and the striatal dopaminergic pathways. Consistent pathological evidence has showed the regional vulnerability of the hippocampus, particularly in the cornu armonis (CA) 2 or CA2/3 exhibiting higher α-synuclein density [12], [17], [18]. This raises questions about the underlying mechanisms through which heterogeneous atrophy of hippocampal subregions caused by PD-related pathologies and striatal dopaminergic pathways interact to affect cognitive function in PD.

Dopaminergic positron emission tomography (PET) imaging provides a noninvasive means to assess the functional integrity of the dopaminergic system [19]. The ^18^F-AV-133 PET tracer has been developed to evaluate the striatal vesicular monoamine transporter type 2 (VMAT2) density of synaptic vesicle in vivo, as a proxy for striatal dopaminergic integrity [20], [21]. Complementarily, MRI offers precise measurements of hippocampal subfield atrophy, including volumetric analysis and asymmetry of hippocampus, which both serve as potentially effective biomarkers for cognitive decline [16]. By using dopaminergic PET and high-resolution structural MRI, it is possible to simultaneously assess striatal dopaminergic dysfunction and hippocampal degeneration, providing a comprehensive understanding of their relationship on cognitive impairment in PD.

This study therefore aims to 1) assess the neurodegeneration of striatal dopamine and hippocampal subfields in PD-NC and PD-MCI, 2) link the degree of neurodegeneration of these regions to neurocognitive domains, and 3) investigate the associations among hippocampal subfield atrophy, striatal dopaminergic degeneration, and cognitive impairment in PD.

## 2 Methods

### 2.1 Participants

Data for the study were obtained from the Parkinson's Progression Markers Initiative (PPMI) cohort, which can be accessed at www.ppmi-info.org/. PPMI is a large, multi-center, and observational study, comprising the comprehensive collection of clinical behavior assessments and imaging outcomes across all stages of PD. All participants included in this study have a confirmed clinical diagnosis of PD, supported by evidence of dopamine transporter (DAT) deficit.

**Fig. 1:**
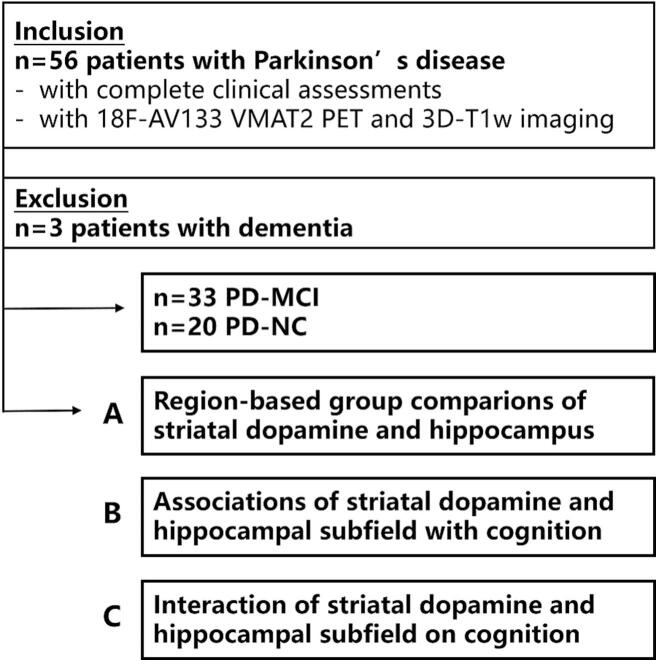
**Study design and flow chart.** Abbreviation: PD = Parkinson’ s disease; PD-MCI = PD with mild cognitive impairment; PD-NC = PD with normal cognition.

From the entire PPMI cohort, 56 PD patients who had complete ^18^F-AV133 VMAT2 PET and MRI scans were selected for inclusion. The time interval between the two imaging scans did not exceed six months. 3 PD patients with dementia were excluded based on the criterion of a Montreal Cognitive Assessment (MoCA) score below 20. In total, 53 PD patients were included in the final analysis (Fig. 1).

### 2.2 Clinical assessments

All the patients underwent a comprehensive battery of motor and cognitive assessments, as detailed in the PPMI database. Motor function was assessed using the Movement Disorder Society-sponsored revision of the Unified Parkinson’s Disease Rating Scale (MDS-UPDRS) Part III and Hoehn-Yahr stages. Cognitive evaluations included the global cognition function using MoCA [22] and specific cognitive domains using a set of neuropsychological batteries. The neuropsychological tests used to assess specific cognitive domains included: (1) Executive function: Symbol Digit Modality Test (SDMT) [23]; (2) Working memory: Letter-Number Sequencing as part of the Wechsler Adult Intelligence Scale-III and Scale-IV test batteries (LNS); (3) Language: 30-item Boston Naming Test (BNT); (4) Episodic memory: Hopkins Verbal Learning Test (HVLT) with delayed recall. PD-MCI was defined according to the MDS MCI task force level I guidelines: cognitive impairment on a scale of global cognitive abilities (MoCA < 26) or impairment on at least 2 neuropsychological tests [24]. Subjects who did not fulfill the above criteria and had normal objective cognition were classified as PD with normal cognition (PD-NC). Finally, 33 PD-MCI and 20 PD-NC were analyzed.

### 2.3 Image acquisition

18F-AV-133 VMAT2 PET scans and T1-weighted MRI scans were acquired at several PPMI imaging centers in accordance with the specified PPMI imaging protocol. The acquisition parameters are detailed in: https://www.ppmi-info.org/study-design/research-documents-and-sops.

### 2.4 Hippocampal subfields volume

Volumetric measures of hippocampal subfields were performed using FreeSurfer (version 6.0) (https://surfer. nmr.mgh.harvard.edu). Automated segmentation of the hippocampal subfields was performed based on a computational atlas of the hippocampal formation using a combination of ex vivo and in vivo MRI data [25]. The atlas includes the hippocampal tail, subiculum, CA1, CA2/3, CA4, the hippocampal fissure, presubiculum, parasubiculum, the granule cell layers of the dentate gyrus (GC-DG), fimbria, the molecular layer (ML), and the hippocampal amygdala transition area (HATA). In addition, to correct the effects of the brain volume size, estimated total intracranial volume (TIV) was calculated for each subject. In this study, we used subfield-to-TIV ratio of volume for further statistical analyses.

### 2.5 PET data analysis, co-registration of PET and MRI images

^18^F-AV-133 VMAT2 PET were processed by two expert radiologists with over 10 years of experience in PET imaging. For cases of discrepancy, the final interpretation was determined by consensus between the two radiologists after an additional review. PET images were preprocessed using Statistical Parametric Mapping 5 (SPM5; Wellcome Trust Centre for Neuroimaging, London, UK; https://www.fil.ion.ucl.ac.uk/spm) implemented in MATLAB R2023b. First, each PET image was rigidly coregistered to the corresponding T1-weighted anatomical MRI using normalized mutual information. Next, tissue segmentation was performed on the T1-weighted image to generate probabilistic maps of gray matter, white matter, and cerebrospinal fluid, which were subsequently used for partial volume effect (PVE) correction. The coregistered PET images were then normalized to the Montreal Neurological Institute (MNI) standard space with an isotropic voxel resolution of 2 mm, using the forward deformation fields derived from tissue segmentation. Following normalization, PET images were smoothed with an isotropic Gaussian kernel of 8 mm full width at half maximum (FWHM). For region-based analysis, the preprocessed PET images were coregistered to the Automated Anatomical Labeling (AAL 116) template.

Under the guidance of co-registration, the occipital lobe was selected as the reference region for volume of interest (VOI) analysis. The bilateral caudate nucleus, bilateral putamen (anterior/posterior portions), and the occipital lobe were manually delineated as VOIs on PET images. LIFEx software (version 6.20, https://www.lifexsoft.org) was used to automatically calculate the average radioactivity count for each VOI [26]. The ^18^F-AV-133 standard uptake volume ratio (SUVR) in these regions was calculated by the formula:.

$$\text{SUVR=}\frac{\text{SUV}\text{meanVOI}}{\text{SUV}\text{meanOccipital}}\;$$

### 2.6 Asymmetry index

To capture interhemispheric imbalance at the individual level, the volumetric asymmetry index (AI) was calculated for both hippocampal subfield volumes and striatal SUVR using the formula:

$$\text{AI=}\frac{\left|\text{Left-Right}\right|}{\left|\text{Left+Right}\right|}\text{×100}$$

We used AI because PD commonly shows asymmetric clinical manifestations and asymmetric nigrostriatal involvement, particularly in non-demented stages. AI therefore provides a subject-level measure of lateralized pathological imbalance while reducing the influence of between-subject differences in absolute regional volume or tracer uptake. Because the present AI formula uses absolute values, larger AI values indicate greater left–right imbalance, whereas smaller AI values indicate greater symmetry, without preserving information about laterality direction.

### 2.7 Statistical analysis

The normal distribution of the data was assessed using the Kolmogorov–Smirnov test. To ensure normally distributed data, AI metrics (hippocampal subfield volumes and striatal subregion SUVR) were square-root or logarithm transformed before statistical analysis.

Demographics and clinical features were compared between PD-MCI and PD-NC, using analysis of two sample t-tests for continuous variables and chi-squared tests for categorical variables.

Partial correlation analyses were conducted to explore associations between AI (hippocampal subfield volumes and striatal SUVR) and cognitive scores in all PD patients, controlling for covariates (including age, sex, and education).

Multivariate linear regression models were fitted to examine the relationship between AI (hippocampal subfield volumes and regional striatal uptake) and cognitive scores. A two-way interaction term (hippocampal subfield volume × regional striatal uptake) were included to assesses whether regional striatum moderates the effect of hippocampus on cognitive decline, in all PD subjects and within diagnostic groups, respectively. Age, sex, and education were considered as covariates in the regression models.

## 3 Results

### 3.1 Demographic and clinical characteristics

The demographic and clinical characteristics of all subjects are summarized in Table1. No significant differences were observed between PD-MCI and PD-NC group in age, sex distribution, or years of education (*p* > 0.05). Additionally, there were no significant differences in MDS-UPDRS III, H-Y stage, disease duration, LEDD, or GDS scores between PD-MCI and PD-NC group (*p* > 0.05). However, PD-MCI group exhibited significantly poorer performance across all cognitive assessments compared to PD-NC group (all p < 0.05), including global cognition (MoCA) and specific domains such as executive function, working memory, and episodic memory.

**Table 1:** Demographic and clinical characteristics of Parkinson’s disease patients.

**Variable**	**PD**		***p* value**
	**PD-MCI** (N = 33)	**PD-NC** (N = 20)	
**Age at onset (y)**	64.780(12.248)	62.667(8.772)	0.468^a^
**Sex (F/M)**	4/16	11/22	0.359^a^
**Education (y)**	16.250(3.144)	16.424(2.927)	0.839^a^
Disease duration (y)*	1.25(1.378)	2.08(1.295)	0.106^b^
**H-Y**	2(0)	2(1)	0.146
**LEDD (mg)**	290.750(288.596)	295.455(185.989)	0.943^a^
**GDS**	5.500(1.147)	5.061(1.248)	0.206^a^
**MDS-UPDRS-Ⅲ**	28.550(11.255)	24.455(10.195)	0.179^a^
MoCA*	24(2.750)	28(3.000)	**<0.001**^b^
**LNS**	8.9(2.36)	11(2.107)	**0.001**^a^
**HVLT-recall**	37.000(11.173)	46.450(10.625)	**0.003**^a^
BNT*	57.5(4.5)	59(2.5)	**0.018**^b^
**SDMT**	38.750(8.045)	46.150(7.878)	**0.002**^a^

Values are expressed as mean (standard deviation) for continuous variables and numbers for gender (female/male). *variables with non-Gaussian distribution are marked with an asterisk, which are expressed as median (interquartile range). Significant statistics results (p < 0.05) are indicated in bold. ^a^ Comparison performed using a parametric test (Student’s *t* test). ^b^ Comparison performed using nonparametric test (Mann–Whitney *U* test).

Abbreviation: PD = Parkinson’s disease; PD-MCI = PD with mild cognitive impairment; PD-NC = PD with normal cognition; H-Y = Hoehn-Yahr stage; LEDD = levodopa equivalent daily dose; MDS-UPDRS-Ⅲ = MDS Unified-Parkinson’ s disease rating scale-Ⅲ; MoCA = Montreal cognitive assessment; LNS = letter-number sequencing; HVLT-recall = Hopkins verbal learning test with delayed recall; SDMT = symbol digit modality test; GDS = geriatric depression scale; BNT = 30-item Boston naming test.

### 3.2 Intergroup differences in hippocampal subfields volumes and striatum regional SUVR

SUVR was calculated for the anterior putamen, posterior putamen, and caudate. The asymmetry indices of hippocampal subfield volumes and striatal SUVR values were presented in Table2**.** While no significant differences were observed in the absolute volumes of hippocampal subfields or striatal SUVR between PD-MCI and PD-NC group (p > 0.05), significant intergroup differences were found in the AI of specific striatal regions, particularly the anterior putamen and caudate (p < 0.05).

**Table 2:** Asymmetry indices of hippocampal subfield volumes and striatal subregion SUVR between PD-MCI and PD-NC.

**Asymmetry indices**	**PD**		***p* value**
	**PD-MCI** (N = 33)	**PD-NC**(N = 20)	
***Hippocampal subfield volumes***			
hippocampal tail	4.805(4.286)	3.855(4.846)	0.541
subiculum	1.428(2.559)	1.726(2.335)	0.652
CA1	2.537(3.395)	2.718(2.406)	0.502
hippocampal fissure	5.705(10.261)	4.637(6.421)	0.451
presubiculum	3.666(5.746)	2.793(6.561)	0.576
parasubiculum	6.067(8.007)	8.380(12.735)	0.119
ML	2.407(4.437)	1.763(1.875)	0.235
GC-DG	3.327(3.718)	2.421(3.418)	0.191
CA2/3	6.908(8.310)	5.402(4.981)	0.093
CA4	3.904(3.666)	2.897(4.001)	0.19
fimbria	12.555(13.537)	6.391(10.264)	0.937
HATA	4.307(3.595)	4.583(5.995)	0.866
whole hippocampus	2.541(3.007)	1.826(1.611)	0.175
***Striatal subregion SUVR***			
caudate	6.433(8.939)	2.297(3.773)	**<0.001**
anterior putamen	5.175(7.714)	2.945(4.667)	**0.039**
posterior putamen	5.222(7.564)	6.463(7.205)	0.885

Values are expressed as median (interquartile range), as asymmetry indices of hippocampal subfields’ volumes and regional SUVR are non-Gaussian distribution variables. Significant statistics results (*p* < 0.05) are highlighted in bold.

Abbreviation: PD = Parkinson’s disease; PD-MCI = PD with mild cognitive decline; PD-NC = PD with normal cognition; CA1/2/3/4 = cornu ammonis 1/2/3/4; GC- DG = granule cell layers of the dentate gyrus; ML = Molecular layer; HATA = hippocampal amygdala transition area; SUVR = standard uptake volume ratio.

### 3.3 Partial correlation analysis of hippocampal subfield volumes, striatal region SUVR, and cognition

Partial correlation analyses, controlling for age, sex, and education, revealed significant associations between asymmetry indices and cognitive performance in the overall PD cohort (Table3).

**Table 3:** The results of partial correlation analysis of asymmetry indices of hippocampal subfields’ volumes, regional SUVR and cognition in the whole PD group.

**Asymmetry indices**	**LNS**			**HVLT-recall**			**BNT**			**SDMT**			**MOCA**	
	r	*p*		r	*p*		r	*p*		r	*p*		r	*p*
hippocampal tail	0.125	0.387		0.158	0.273		0.182	0.206		−0.052	0.719		−0.075	0.605
subiculum	−0.137	0.342		−0.095	0.511		0.011	0.938		−0.098	0.499		−0.042	0.773
CA1	0.203	0.157		0.004	0.979		0.093	0.52		−0.032	0.827		−0.123	0.396
hippocampal fissure	0.059	0.683		0.041	0.775		−0.19	0.187		0.191	0.185		−0.021	0.882
presubiculum	−0.083	0.566		−0.016	0.91		−0.006	0.969		0.152	0.293		0.075	0.603
parasubiculum	0.107	0.461		0.168	0.243		−0.249	0.081		0.101	0.487		0.274	0.054
ML	0.089	0.541		−0.179	0.214		0.076	0.601		−0.129	0.372		−0.146	0.31
GC-DG	−0.019	0.894		−0.217	0.131		−0.055	0.702		−0.184	0.201		**−0.282**	**0.047**
CA2/3	−0.031	0.83		**−0.323**	**0.022**		−0.052	0.719		−0.113	0.436		**−0.285**	**0.045**
CA4	0.003	0.983		−0.174	0.227		−0.071	0.626		−0.17	0.237		−0.276	0.052
fimbria	0.1	0.49		−0.011	0.942		0.052	0.72		−0.017	0.907		−0.033	0.82
HATA	0.083	0.568		0.053	0.717		0.156	0.279		−0.06	0.677		0.108	0.456
whole hippocampus	0.147	0.308		−0.225	0.116		0.155	0.283		−0.226	0.114		−0.225	0.116
caudate	**−0.428**	**0.002**		**−0.35**	**0.013**		**−0.342**	**0.015**		−0.267	0.061		**−0.417**	**0.003**
anterior putamen	**−0.332**	**0.019**		**−0.507**	**<0.001**		−0.083	0.568		**−0.325**	**0.021**		**−0.362**	**0.01**
posterior putamen	**−0.29**	**0.041**		0.149	0.301		**−0.289**	**0.042**		−0.188	0.192		−0.257	0.071

r = correlation coefficient. Significant statistics results (*p* < 0.05) are indicated in bold.

Abbreviation: CA1/2/3/4 = cornu ammonis 1/2/3/4; GC-DG = granule cell layers of the dentate gyrus; HATA = hippocampal amygdala transition area; ML = molecular layer; SUVR = standard uptake volume ratio; MoCA = Montreal cognitive assessment; LNS = letter-number sequencing; HVLT-recall = Hopkins verbal learning test with delayed recall; SDMT = symbol digit modality test.

No significant associations were observed between the absolute volumes of hippocampal subfields or striatal SUVR and cognitive scores in the overall PD group. However, the AI of CA2/3 vol was negatively correlated with MoCA (p = 0.045, r = -0285) and HVLT with delayed recall scores (p = 0.022, r = -0.323). Similarly, the AI of GC-DG was negatively associated with HVLT with delayed recall scores (p = 0.047, r = -0.282).

The AI of striatal SUVR regions showed stronger associations with cognitive scores. The AI of caudate SUVR was negatively correlated with LNS (p = 0.002, r = -0.482), HVLT with delayed recall (p = 0.013, r = -0.35), BNT (p = 0.015, r = -0.342), and MoCA scores (p = 0.003, r = -0.417). The AI of anterior putamen SUVR was associated with LNS (p = 0.019, r = -0.332), HVLT with delayed recall (p < 0.001, r = -0.507), SDMT (p = 0.021, r = -0.325), and MoCA scores (p = 0.01, r = -0.362). The AI of posterior putamen SUVR was linked to LNS (p = 0.041, r = -0.29) and BNT scores (p = 0.042, r = -0.289).

### 3.4 Multivariate linear regression models

Multivariate linear regression models were used to explore the interaction effects between hippocampal subfield volumes and striatal SUVR on cognitive performance in the whole PD group and subgroups (Table4**)**. Variables that showed significant correlations in the partial correlation analysis were further included in the regression models.

**Table 4:** The results of multiple linear regression models investigating the interaction of hippocampal volumes and striatum SUR on cognition in the whole PD group.

**Variables**	**Model 1: MoCA**			
	**Unstandardized β**	**Standardized β**	**t**	***p***
Sex	−1.345	−0.22	−1.583	0.12
Education	−0.021	−0.023	−0.169	0.867
Age	0.017	0.061	0.43	0.669
CA2/3 AI	0.01	0.003	0.017	0.986
Anterior putamen AI	2.365	0.089	0.374	0.71
CA2/3 AI × Anterior putamen AI	−0.652	−0.57	−2.127	**0.039**
*F*	3.691			
*p*	**0.004**			
**Variables**	**Model 2: HVLT-recall**			
	**Unstandardized β**	**Standardized β**	**t**	***p***
Sex	−0.564	−0.022	−0.168	0.867
Education	1.146	0.292	2.288	**0.027**
Age	−0.19	−0.165	−1.19	0.24
CA2/3 AI	−12.94	−0.892	−2.46	**0.018**
Caudate AI	−13.352	−1.351	−2.481	**0.017**
CA2/3 AI × Caudate AI	10.281	1.364	1.985	0.053
*F*	3.499			
*p*	**0.006**			
	**Model 3: HVLT-recall**			
**Variables**	**Unstandardized β**	**Standardized β**	**t**	***p***
Sex	−4.197	−0.163	−1.185	0.242
Education	1.229	0.314	2.493	**0.016**
Age	−0.048	−0.042	−0.312	0.756
GC-DG AI	−82.422	−0.737	−2.587	**0.013**
Anterior putamen AI	−3.87	−0.259	−1.142	0.259
GC-DG AI × Anterior putamen AI	2.49	0.395	1.097	0.278
*F*	3.726			
*p*	**0.004**			

Significant statistics results (*p* < 0.05) are indicated in bold.

Abbreviation: β = model coefficient; AI = asymmetry indices; CA2/3 = cornu ammonis 2/3; GC-DG = granule cell layers of the dentate gyrus; MoCA = Montreal cognitive assessment; HVLT-recall = Hopkins verbal learning test with delayed recall.

In the overall PD cohort, the AI of CA2/3 volumes and the AI of anterior putamen SUVR demonstrated significant interaction effect on MoCA scores (p = 0.039, stβ = -0.57). This indicated that the association between greater CA2/3 asymmetry and worse global cognition was stronger in patients with lower anterior putamen SUVR asymmetry and weaker in patients with higher anterior putamen SUVR asymmetry. In other words, the relationship between CA2/3 asymmetry and cognition varied according to the degree of anterior putamen dopaminergic asymmetry. Additionally, the AI of CA2/3 volumes and AI of caudate SUVR independently predicted HVLT with delayed recall scores (CA2/3: p = 0.018, stβ = -0.892; caudate SUVR: p = 0.017, stβ = -1.351), though no significant interaction was observed between these variables.

In the PD-MCI subgroup, a significant interaction was found between the AI of CA2/3 vol and the AI of posterior putamen SUVR on MoCA scores (p = 0.002, stβ = -2.75) (Table5), suggesting that the association between CA2/3 asymmetry and global cognition also varied with posterior putamen dopaminergic asymmetry in cognitively impaired patients.

**Table 5:** The results of multiple linear regression models investigating the interaction of hippocampal volumes and striatum SUR on cognition in the PD-MCI group.

**Variables**	**Model 1: MoCA**			
	**Unstandardized β**	**Standardized β**	**t**	***p***
Sex	1.379	0.297	1.082	0.299
Education	−0.054	−0.089	−0.456	0.656
Age	0.006	0.04	0.139	0.892
CA2/3 AI	2.558	1.189	3.277	**0.006**
Posterior putamen AI	29.191	1.962	2.917	**0.012**
CA2/3 AI × Posterior putamen AI	−13.169	−2.75	−3.8	**0.002**
*F*	4.112			
*p*	**0.015**			

Significant statistics results (*p* < 0.05) are indicated in bold.

Abbreviation: β = model coefficient; AI = asymmetry indices; CA2/3 = cornu ammonis 2/3; MoCA = Montreal cognitive assessment.

No significant interactions were found between AI of hippocampal subfield volumes and AI of striatal SUVR in PD-NC group.

## 4 Discussion

In the current work, we investigated the roles of nigrostriatal dopamine dysfunction and hippocampal subfield atrophy in cognitive decline in PD using VMAT2 PET and high-resolution structural MRI. Our findings revealed the interaction between hippocampal CA2/3 asymmetry and anterior putamen dopaminergic deficits in cognitive impairment in PD. We also found an interaction effect on cognitive decline between hippocampal CA2/3 asymmetry and posterior putamen in PD-MCI.

Nigrostriatal dopamine and hippocampus were collectively considered as the critic contributors to cognitive impairment in PD. In this study, not only do we provide in vivo evidence of the correlation of hippocampal atrophy and striatal dopaminergic deficits with cognition, but also interaction between hippocampal CA2/3 subfield asymmetry and anterior putamen dopaminergic deficits asymmetry in cognitive decline in PD. This interaction demonstrated that reduced asymmetry in anterior putamen dopaminergic integrity was associated with a stronger negative impact of CA2/3 asymmetry on global cognition, suggesting that bilateral dopaminergic denervation in anterior putamen may amplify the damaging effect of hippocampal atrophy on cognitive decline in PD. In contrast, increased asymmetry of anterior putamen dopaminergic integrity was related to a weaker negative effect of CA2/3 asymmetry on global cognition, indicating that preserved anterior putamen asymmetry may attenuate this negative effect of hippocampus on cognition. These findings support a cooperative model of hippocampal-striatal interaction, which proposed that hippocampal and striatal activities are coordinated and work in parallel for cognition rather than competing [27]. This also aligns with animal studies showing coordinated hippocampal-dorsomedial striatal activity in memory tasks [28]. Furthermore, a task-based functional MRI research suggested compensatory hippocampal activation in PD-NC, which was absent in PD-MCI [15], [16]. Therefore, we assumed that this hippocampal-striatal interaction may exhibit a compensatory mechanism when ipsilateral striatal dopaminergic is preserved in cognitively intact PD patients, and the compensatory response reduced after bilateral striatal dopaminergic denervation. Our results further extend the cooperative model of hippocampal-striatal interaction to PD pathophysiology underlying cognitive impairment, and emphasize the role of dopaminergic loss in modulating hippocampal structural changes in cognitive decline in PD.

The unique interaction between CA2/3 asymmetry and striatal dopaminergic deficits in driving cognitive impairment aligns with neuropathological evidence identifying CA2/3 as a selective site of α-synuclein aggregation in PD, distinct from adjacent hippocampal subfields that remain relatively spared [12], [29], [30], [31]. While imaging studies have linked volume loss in other subfields (e.g., CA1, subiculum) to PD-MCI progression [32], [33], our findings reveal that CA2/3 asymmetry specifically interacts with anterior putamen dopaminergic dysfunction to exacerbate cognitive decline. The unique interaction in CA2/3 suggest that α-synuclein accumulation in CA2/3 may disrupt hippocampal-striatal circuitry by abnormal excitability and impaired synaptic transmission [13], and concurrent dopaminergic denervation in the anterior putamen amplifies this disruption. Importantly, no such interactions were observed for other hippocampal subregions (e.g., GC-DG, subiculum), underscoring the unique vulnerability of CA2/3 in early cognitive impairment in PD.

Asymmetry of dopaminergic deficiency in the caudate and anterior putamen were higher in PD-MCI than PD-NC, and these were also associated with multidomain cognitive deficits. These observations align with PET studies linking caudate and anterior putamen dopamine depletion to dementia conversion [34]. Notably, posterior putamen SUVR showed no independent association with MoCA scores, consistent with its predominant role in motor progression rather than cognition [35]. However, the interaction between posterior putamen SUVR asymmetry and CA2/3 asymmetry in PD-MCI seen in our study suggest a non-linear association between posterior putamen dopamine and cognition, depending on the interactive association between posterior putamen dopaminergic deficits and hippocampus subfield.

Hippocampal asymmetry has been considered as a potentially effective biomarker for predicting the risk of AD [36], but little is known about the asymmetry of hippocampal subfield volumes in the development of dementia in PD. In our study, the association of CA2/3 and GC-DG asymmetry with global cognition or episodic memory indicate their contribution to episodic memory decline and dementia progression in PD. We failed to discover associations between hippocampal volumes and cognitive assessments in PD, despite prior studies indicating hippocampal volume loss in PD-MCI [32], [33]. A voxel-based *meta*-analysis investigating gray matter volume associated with cognitive impairment in PD reported that hippocampal gray matter atrophy may not be an early marker of cognitive decline in PD, but a sign for the progression from PD-MCI to PD dementia [37]. Instead, the lateralized atrophy in the CA2/3 and GC-DG, rather than absolute volumetric measures, may serve as a more sensitive marker of cognitive impairment in PD.

Several important limitations should be acknowledged in the study. First, the study included only PD-MCI and PD-NC patients, and excluded those with dementia due to insufficient sample size. Future research should examine whether the observed interactions extend to PD dementia. Second, despite the use of ultra-high-resolution MRI, certain hippocampal subregion boundaries remain difficult to delineate, such as the interfaces between the CA fields along the hippocampal pyramidal layer or the CA4/GC–DG boundary. This continues to be an unresolved issue in hippocampal subregion MRI research, where there is significant variation and inconsistency in how subregions are defined [25]. Moreover, the sample size in the study was relatively small, as we included patients who underwent both ^18^F-AV133 VMAT2 PET and MRI scans within a six-month interval. The PD-NC subgroup was particularly limited. Although the study could have benefitted from a larger sample, especially by including data from additional radiolabeled dopaminergic PET scans (e.g., dopamine synthesis [AADC activity] and transport [DAT availability]) from the PPMI cohort, the use of VMAT2 imaging minimizes confounding from pharmacological interventions [20], [38], offering a stable marker for longitudinal monitoring [39]. Future studies should incorporate larger, independent cohorts and additional dopaminergic PET modalities to validate these findings. It should be noted that FDR correction was applied for multiple comparisons in this analysis; however, the corrected results did not remain significant. The reported p-values should therefore be interpreted as preliminary and exploratory and considered in the context of the effect sizes and biological plausibility. Finally, this study did not evaluate whether motor symptom severity (such as MDS-UPDRS III scores or Hoehn-Yahr stage) moderates the relationships among hippocampal asymmetry, striatal asymmetry, and cognitive outcomes. Given the well-established role of dopaminergic dysfunction in both motor and non-motor manifestations of Parkinson’s disease, the absence of motor severity as a moderator in the present analyses limits the completeness of clinical interpretation. Future studies with larger sample sizes are warranted to systematically examine how motor status may influence these hippocampal-striatal interactions.

## 5 Conclusion

The study combined striatal dopaminergic dysfunction with hippocampal subfield atrophy to investigate their association on cognitive function in PD. The findings revealed that both striatal dopaminergic asymmetry and hippocampal subfield asymmetry are associated with cognitive impairment in PD, and the association between CA2/3 asymmetry and global cognition may vary according to the degree of striatal dopaminergic asymmetry. These results provided a deeper insight into the neural mechanisms underlying cognitive impairment in PD.

## 6 Ethical approval and Informed consent statement

PPMI is a large multicenter study and each site independently received ethics approval of the protocol. All subjects gave written informed consent in accordance with the Declaration of Helsinki. See Marek et al. (2011) and the online PPMI protocol for details of the PPMI study and image acquisition, respectively. Ethics committee approval and individual patient consents were received by the PPMI (https://www.ppmi-info.org/study-design/research-documents-and-sops). The present study was approved by the second affiliated hospital of Zhejiang university school of medicine.

## 7 Data availability Statement

The data that support the findings of this study are available from the corresponding author upon reasonable request.

## CRediT authorship contribution statement

**Jingjing Xu:** Writing – review & editing, Writing – original draft, Supervision, Software, Resources, Project administration, Methodology, Investigation, Funding acquisition, Formal analysis, Data curation, Conceptualization. **Sijia Tan:** Writing – review & editing, Writing – original draft, Supervision, Software, Resources, Project administration, Methodology, Investigation, Formal analysis, Data curation, Conceptualization. **Xiaohui Zhang:** Writing – review & editing, Methodology, Formal analysis, Data curation, Conceptualization. **Minming Zhang:** Writing – review & editing, Resources, Project administration, Investigation. **Xiaojun Xu:** Writing – review & editing, Writing – original draft, Project administration, Methodology, Investigation, Formal analysis, Data curation, Conceptualization.

## Funding

This work was supported by the National Natural Science Foundation of China (Grant No. 82202089). PPMI − a public–private partnership − is funded by the Michael J. Fox Foundation for Parkinson's Research and funding partners, including a consortium of industry players, non-profit organizations and private individuals. Corporate funding partners include AbbVie, Avid, Biogen, Bristol-Myers Squibb, General Electric Healthcare, BioLegend, Genentech, GlaxoSmithKline, Lilly, Lundbeck, Merck, Meso Sale Discovery, Pfizer, Piramal, Roche, Sanofi Genzyme, Servier, Takeda, TEVA, and Union Chimique Belge, and philanthropic funding partner include GOLUB CAPITAL.

## Declaration of competing interest

The authors declare that they have no known competing financial interests or personal relationships that could have appeared to influence the work reported in this paper.

## References

[1] McGregor M.M., Nelson A.B. (2019). Circuit mechanisms of Parkinson's disease. Neuron.

[2] A.J. Yarnall, D.P. Breen, G.W. Duncan, T.K. Khoo, S.Y. Coleman, M.J. Firbank, C. Nombela, S. Winder-Rhodes, J.R. Evans, J.B. Rowe, B. Mollenhauer, N. Kruse, G. Hudson, P.F. Chinnery, J.T. O'Brien, T.W. Robbins, K. Wesnes, D.J. Brooks, R.A. Barker, D.J. Burn; ICICLE-PD Study Group. Characterizing mild cognitive impairment in incident Parkinson disease: the ICICLE-PD study. Neurology. 82(4) (2014) 308-316. https://doi.org/10.1212/wnl.0000000000000066. 10.1212/WNL.0000000000000066 PMC3929202 24363137

[3] Janvin C.C., Larsen J.P., Aarsland D., Hugdahl K. (2006). Subtypes of mild cognitive impairment in Parkinson's disease: progression to dementia. Mov. Disord..

[4] Citro S., Lazzaro G.D., Cimmino A.T., Giuffre G.M., Marra C., Calabresi P. (2024). A multiple hits hypothesis for memory dysfunction in Parkinson disease. Nat. Rev. Neurol..

[5] Rinne J.O., Portin R., Ruottinen H., Nurmi E., Bergman J., Haaparanta M., Solin O. (2000). Cognitive impairment and the brain dopaminergic system in Parkinson disease: [18F]fluorodopa positron emission tomographic study. Arch. Neurol..

[6] Calabresi P., Castrioto A., Di Filippo M., Picconi B. (2013). New experimental and clinical links between the hippocampus and the dopaminergic system in Parkinson's disease. Lancet Neurol..

[7] Lisman J.E., Grace A.A. (2005). The hippocampal-VTA loop: controlling the entry of information into long-term memory. Neuron.

[8] Kobayashi K., Suzuki H. (2007). Dopamine selectively potentiates hippocampal mossy fiber to CA3 synaptic transmission. Neuropharmacology.

[9] Goto Y., Grace A.A. (2005). Dopamine-dependent interactions between limbic and prefrontal cortical plasticity in the nucleus accumbens: disruption by cocaine sensitization. Neuron.

[10] Wittmann B.C., Schott B.H., Guderian S., Frey J.U., Heinze H.J., Düzel E. (2005). Reward-related FMRI activation of dopaminergic midbrain is associated with enhanced hippocampus-dependent long-term memory formation. Neuron.

[11] Goedert M., Spillantini M.G., Del Tredici K., Braak H. (2013). 100 years of Lewy pathology. Nat. Rev. Neurol..

[12] H. Braak, K. Del Tredici, U. Rüb, R.A. de Vos, E.N. Jansen Steur, E. Braak. Staging of brain pathology related to sporadic Parkinson's disease. Neurobiol Aging. 24(2) (2003) 197-211. https://doi.org/10.1016/s0197-4580(02)00065-9. 10.1016/s0197-4580(02)00065-9 12498954

[13] Diógenes M.J., Dias R.B., Rombo D.M., Miranda H.V., Maiolino F., Guerreiro P., Näsström T., Franquelim H.G., Oliveira L.M.A., Castanho M.A.R.B., Lannfelt L., Bergström J., Ingelsson M., Quintas A., Sebastião A.M., Lopes L.V., Outeiro T.F. (2012). Extracellular alpha-synuclein oligomers modulate synaptic transmission and impair LTP via NMDA-receptor activation. J. Neurosci..

[14] Hall H., Reyes S., Landeck N., Bye C., Leanza G., Double K., Thompson L., Halliday G., Kirik D. (2014). Hippocampal Lewy pathology and cholinergic dysfunction are associated with dementia in Parkinson's disease. Brain.

[15] Nagano-Saito A., Habak C., Mejía-Constaín B., Degroot C., Monetta L., Jubault T., Bedetti C., Lafontaine A.L., Chouinard S., Soland V., Ptito A., Strafella A.P., Monchi O. (2014). Effect of mild cognitive impairment on the patterns of neural activity in early Parkinson's disease. Neurobiol. Aging.

[16] Pourzinal D., Yang J.H.J., Bakker A., McMahon K.L., Byrne G.J., Pontone G.M., Mari Z., Dissanayaka N.N. (2021). Hippocampal correlates of episodic memory in Parkinson's disease: a systematic review of magnetic resonance imaging studies. J. Neurosci. Res..

[17] Pereira J.B., Junque C., Bartres-Faz D., Ramirez-Ruiz B., Marti M.J., Tolosa E. (2013). Regional vulnerability of hippocampal subfields and memory deficits in Parkinson's disease. Hippocampus.

[18] Flores-Cuadrado A., Ubeda-Banon I., Saiz-Sanchez D., de la Rosa-Prieto C., Martinez-Marcos A. (2016). Hippocampal alpha-synuclein and interneurons in Parkinson's disease: data from human and mouse models. Mov. Disord..

[19] Yang Y., Li X., Lu J., Ge J., Chen M., Yao R., Tian M., Wang J., Liu F., Zuo C. (2025). Recent progress in the applications of presynaptic dopaminergic positron emission tomography imaging in parkinsonism. Neural Regen. Res..

[20] Hefti F.F., Kung H.F., Kilbourn M.R., Carpenter A.P., Clark C.M., Skovronsky D.M. (2010). (18)F-AV-133: a selective VMAT2-binding radiopharmaceutical for pet imaging of dopaminergic neurons. PET Clin..

[21] Villemagne V.L., Okamura N., Pejoska S., Drago J., Mulligan R.S., Chételat G., Ackermann U., O'Keefe G., Jones G., Gong S., Tochon-Danguy H., Kung H.F., Masters C.L., Skovronsky D.M., Rowe C.C. (2011). In vivo assessment of vesicular monoamine transporter type 2 in dementia with lewy bodies and Alzheimer disease. Arch. Neurol..

[22] Li X., Jia S., Zhou Z., Jin Y., Zhang X., Hou C., Zheng W., Rong P., Jiao J. (2018). The role of the montreal cognitive assessment (MoCA) and its memory tasks for detecting mild cognitive impairment. Neurol. Sci..

[23] Sandry J., Simonet D.V., Brandstadter R., Krieger S., Sand I.K., Graney R.A., Buchanan A.V., Lall S., Sumowski J.F. (2021). The Symbol Digit Modalities Test (SDMT) is sensitive but non-specific in MS: Lexical access speed, memory, and information processing speed independently contribute to SDMT performance. Mult. Scler. Relat. Disord..

[24] Litvan I., Goldman J.G., Tröster A.I., Schmand B.A., Weintraub D., Petersen R.C., Mollenhauer B., Adler C.H., Marder K., Williams-Gray C.H., Aarsland D., Kulisevsky J., Rodriguez-Oroz M.C., Burn D.J., Barker R.A., Emre M. (2012). Diagnostic criteria for mild cognitive impairment in Parkinson's disease: movement disorder society task force guidelines. Mov. Disord..

[25] Iglesias J.E., Augustinack J.C., Nguyen K., Player C.M., Player A., Wright M., Roy N., Frosch M.P., McKee A.C., Wald L.L., Fischl B., K. (2015). Van Leemput; Alzheimer's disease neuroimaging initiative. a computational atlas of the hippocampal formation using ex vivo, ultra-high resolution MRI: application to adaptive segmentation of in vivo MRI. Neuroimage.

[26] Nioche C., Orlhac F., Boughdad S., Reuzé S., Goya-Outi J., Robert C., Pellot-Barakat C., Soussan M., Frouin F., Buvat I. (2018). LIFEx: a freeware for radiomic feature calculation in multimodality imaging to accelerate advances in the characterization of tumor heterogeneity. Cancer Res..

[27] Poldrack R.A., Packard M.G. (2003). Competition among multiple memory systems: converging evidence from animal and human brain studies. Neuropsychologia.

[28] Delcasso S., Huh N., Byeon J.S., Lee J., Jung M.W., Lee I. (2014). Functional relationships between the hippocampus and dorsomedial striatum in learning a visual scene-based memory task in rats. J. Neurosci..

[29] Harding A.J., Halliday G.M. (2001). Cortical Lewy body pathology in the diagnosis of dementia. Acta Neuropathol..

[30] Kalaitzakis M.E., Christian L.M., Moran L.B., Graeber M.B., Pearce R.K., Gentleman S.M. (2009). Dementia and visual hallucinations associated with limbic pathology in Parkinson's disease. Parkinsonism Relat. Disord..

[31] Churchyard A., Lees A.J. (1997). The relationship between dementia and direct involvement of the hippocampus and amygdala in Parkinson's disease. Neurology.

[32] Foo H., Mak E., Chander R.J., Ng A., Au W.L., Sitoh Y.Y., Tan L.C.S., Kandiah N. (2017). Associations of hippocampal subfields in the progression of cognitive decline related to Parkinson's disease. Neuroimage Clin..

[33] Low A., Foo H., Yong T.T., Tan L.C.S., Kandiah N. (2019). Hippocampal subfield atrophy of CA1 and subicular structures predict progression to dementia in idiopathic Parkinson's disease. J. Neurol. Neurosurg. Psychiatry.

[34] S.J. Chung, H.S. Lee, H.S. Yoo, Y.H. Lee, P.H. Lee, Y.H. Sohn. Patterns of striatal dopamine depletion in early Parkinson disease: Prognostic relevance. Neurology. 95(3) (2020) e 280-290. https://doi.org/10.1212/wnl.0000000000009878. 10.1212/WNL.0000000000009878 32616674

[35] Jeong S.H., Park C.W., Lee H.S., Kim Y.J., Yun M., Lee P.H., Sohn Y.H., Chung S.J. (2023). Patterns of striatal dopamine depletion and motor deficits in de novo Parkinson's disease. J. Neural Transm. (Vienna).

[36] Sarica A., Vasta R., Novellino F., Vaccaro M.G., Cerasa A., Quattrone A. (2018). Alzheimer's disease neuroimaging initiative. MRI asymmetry index of hippocampal subfields increases through the continuum from the mild cognitive impairment to the alzheimer's disease. Front. Neurosci..

[37] Y. Xu, J. Yang, X. Hu, H. Shang. Voxel-based meta-analysis of gray matter volume reductions associated with cognitive impairment in Parkinson's disease. J Neurol. 263(6) (2016) 1178-1187.https://doi.org/10.1007/s00415-016-8122-3. 10.1007/s00415-016-8122-3 27113603

[38] T. Vander Borght, M. Kilbourn, T. Desmond, D. Kuhl, K. Frey. The vesicular monoamine transporter is not regulated by dopaminergic drug treatments. Eur J Pharmacol. 294(2-3) (1995) 577-583. https://doi.org/10.1016/0014-2999(95)00594-3. 10.1016/0014-2999(95)00594-3 8750721

[39] L.C. Beauchamp, V. Dore, V.L. Villemagne, S. Xu, D. Finkelstein, K.J. Barnham, Christopher Rowe. Using (18)F-AV-133 VMAT2 PET Imaging to Monitor Progressive Nigrostriatal Degeneration in Parkinson Disease. Neurology. 101(22) (2023) e 2314-2324. https://doi.org/10.1212/wnl.0000000000207748. 10.1212/WNL.0000000000207748 PMC10727223 37816639

